# *Parelaphostrongylus tenuis* is newly endemic in white-tailed deer (*Odocoileus virginianus*) in central Saskatchewan

**DOI:** 10.1016/j.ijppaw.2026.101196

**Published:** 2026-01-21

**Authors:** Linnea McLellan, Trent K. Bollinger, Gabrielle Large, Phil McLoughlin, Erin Moffatt, Louwrens P. Snyman, Iga Stasiak, Maarten Voordouw, Emily Jenkins

**Affiliations:** a Department of Veterinary Microbiology, Western College of Veterinary Medicine, University of Saskatchewan, Saskatoon, Canada; b Department of Veterinary Pathology, Western College of Veterinary Medicine, University of Saskatchewan, Saskatoon, Canada; c Department of Biology, University of Saskatchewan, Saskatoon, Canada; d Saskatchewan Ministry of Environment, Saskatoon, Canada; e Western/Northern Region, Canadian Wildlife Health Cooperative, Western College of Veterinary Medicine, Saskatoon, Canada; f Royal Alberta Museum, Edmonton, Canada

## Abstract

*Parelaphostrongylus tenuis*, or meningeal worm, can be fatal and have detrimental effects at a population level in cervid species outside of the definitive host, the white-tailed deer (WTD) (*Odocoileus virginianus*), in which they cause little or no ill effects. Although well documented in Canada east of Saskatchewan, there is only one published case of *P. tenuis* in WTD in Saskatchewan from the 1990s, representing the northwestern distributional limit of this parasite in North America. We report 90 confirmed (47) or suspected (43) cases of *P. tenuis* diagnosed in natural mortalities in moose (*Alces alces*), elk (*Cervus canadensis*), and mule deer (*Odocoileus hemionus*) by the Western/Northern region of the Canadian Wildlife Health Cooperative between 2015 and 2024. To determine if this parasite was locally acquired, we examined meninges and associated venous sinuses of 330 WTD harvested in fall/winters of 2021–2024. We detected a mean of 2 (1–10) adult *P. tenuis* in 10.6 % of sampled WTD from 16 Wildlife Management Zones across central and northeastern SK, much further west than previously recorded. There was substantial overlap between endemic zones for the parasite in WTD and cases in aberrant hosts, suggesting local transmission. DNA was extracted from 4 nematodes from 4 separate infected WTD for each of the sampling years (16 worms) and assayed with PCR using primers targeting the Cytochrome C Oxidase Subunit I (COI) of mitochondrial DNA. Sequences for COI had 99.8 % similarity to sequences of *P. tenuis* in GenBank, and were most similar to sequences from Manitoba, Canada. Our results demonstrate establishment of viable populations of *P. tenuis* in WTD in east-central Saskatchewan, evidence of spillover into aberrant hosts, and the potential for both further westward and northward expansion, which could pose a risk to threatened boreal caribou (*Rangifer tarandus*) and moose populations. This work informs wildlife managers of a new threat cervid populations may face in western and northern North America.

## Highlights

• *Parelaphostrongylus tenuis* linked to death in aberrant cervid hosts.
• Adult nematodes found in white-tailed deer (WTD) definitive hosts harvested 2021-24.
• Highlights local transmission and spillover from WTD into other wild cervids.
• *P. tenuis* known western distributional limit now established in central Saskatchewan.

## Graphical abstract

**Figure:**
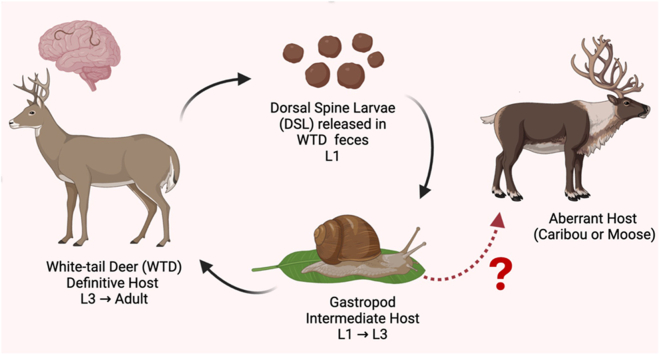

## 1 Introduction

Meningeal worm (*Parelaphostrongylus tenuis*) is highly pathogenic in several cervid species and therefore of potential concern to researchers, hunters, producers, and Indigenous stakeholders. Carried by white-tailed deer (*Odocoileus virginianus*, WTD), their definitive hosts, meningeal worm has long been recognized in eastern Canada over the last century but was thought to be largely excluded from regions west of Manitoba (Bindernagel and Anderson, 1972; Wasel et al., 2003). The parasite is thought to be a contributing factor to decline in moose populations across several eastern Canadian provinces and may pose a risk to populations further north, possibly precipitated by northward movement of WTD (Feldman et al., 2017). Historically, failed caribou reintroduction efforts in eastern provinces have been suspected to be in part a result of *P. tenuis* transmission in habitat shared with WTD (Dauphine, 1975). While WTD typically demonstrate no pathology or clinical disease from this parasite, aberrant hosts such as caribou (*Rangifer tarandus*), moose (*Alces alces*), elk (*Cervus canadensis*), mule deer (*Odocoileus hemionus*), and pronghorn antelope (*Antilocapra americana*) can develop severe disease and often succumb to the infection quickly (Lankester, 2001; Duffy et al., 2004). *Parelaphostrongylus tenuis* poses a similar risk to some livestock species in the camelid family such as camels (*Camelus* spp.), llamas (*Lama glama*), and alpacas (*Vicugna pacos*), as well as members of the Bovidae family such as sheep (*Ovis aries*), and goats (*Capra aegagrus hircus*), while cattle (*Bos taurus*) are rarely clinically affected (Mitchell et al., 2011).

**Fig. 1:**
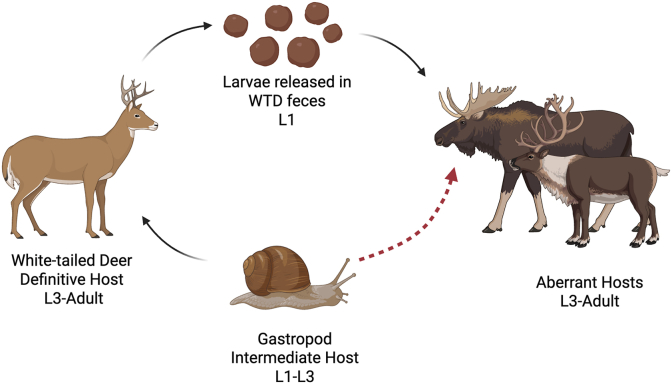
Life cycle of *Parelaphostrongylus tenuis*. First stage larvae (L1) are released in the feces of white-tailed deer (WTD, *Odocoileus virginianus*) and invade a terrestrial gastropod intermediate host where they develop to third stage larvae (L3). When the gastropod is consumed by a WTD, L3 develop into adult worms. When the gastropod is consumed by an aberrant host, the infected animal often dies before L1 are shed, in the prepatent period. Created with Biorender.com.

**Fig. 2:**
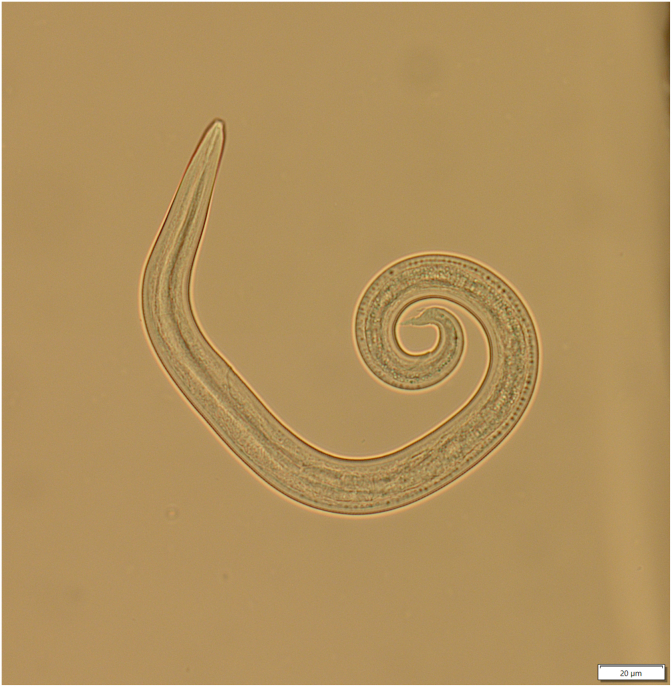
Dorsal spined larvae (DSL) recovered from cervid feces, which are produced by several protostrongylid genera, including *Parelaphostrongylus* and *Varestrongylus* spp. in North America.

Adult *P. tenuis* live within the meninges of WTD (Fig. 1) but migrate through the nervous tissue of the brain and spinal cord in aberrant hosts (Wasel et al., 2003; Verocai et al., 2020). Cervids other than WTD infected with *P. tenuis* often die from the subadult worms before infections become patent (Lankester, 2001). Many protostrongylids (including all species of *Parelaphostrongylus*) shed morphologically similar first stage larvae with distinct spined tails, earning them the name Dorsal Spined Larvae (DSL) (Fig. 2) (Kafle et al., 2017). After DSL are released in WTD feces, larvae develop within infected terrestrial gastropods into the third larval stage (Fig. 1), infective to deer and other cervids (Platt, 1989). Several taxa of gastropods can serve as intermediate hosts for *P. tenuis*, suggesting that the disease distribution is less likely to be limited by gastropod species distribution, but rather by ecological factors impacting gastropod population dynamics (Wasel et al., 2003) and abundance (Severud et al., 2023). The drier regions of Canada's southern prairies are thought to act as a possible barrier to the western movement of the disease due to lower densities of gastropod populations (Wasel et al., 2003). However, related protostrongylids such as *P. andersoni* and *Varestrongylus alpenae*, which have a similar life cycle, have been reported in Saskatchewan (Gray et al., 1985; Lankester and Fong, 1989; Verocai et al., 2020).

A major limitation to non-invasive surveys for *P. tenuis* is that multiple protostrongylid species produce nearly identical DSL in the feces of cervid hosts. The protostrongylid nematodes *Parelaphostrongylus andersoni* and *Varestrongylus eleguneniensis* have been found in caribou and moose in northern and central Canada (S. Kutz, 2001; S. J. Kutz et al., 2007; Verocai et al., 2014; Bondo et al., 2019; Verocai et al., 2020; Pidwerbesky et al., 2023) and *V. alpenae,* a lungworm, has been commonly found in WTD in Saskatchewan (Gray et al., 1985; Kutz et al., 2007). These species share the same protostrongylid life cycle as *P. tenuis* (Fig. 1) but are much less pathogenic, ​as adults are found primarily in muscle or lung tissue, depending on the species (Asmundsson et al., 2008; Verocai et al., 2020; Pidwerbesky et al., 2023)​. *Parelaphostrongylus andersoni* infection is primarily associated with caribou in more northern latitudes and may spill over into sympatric moose populations (Lankester and Fong, 1989; Lankester, 2001; Verocai et al., 2020). White-tail deer in North America are also suitable definitive hosts for *P. andersoni* and this parasite may be more widespread across WTD distribution ranges than previously recorded (Prestwood, 1972; Lankester and Fong, 1989; Lankester, 2001; Asmundsson et al., 2008). Previous studies had found that coinfections of both *P. andersoni* and *P. tenuis* were uncommon (Lankester and Fong, 1989); however, these studies did not have access to molecular detection methods, and thus co-infection rates may have been under-reported.

Due to the difficulty to identify DSL to species level, surveys for *P. tenuis* have relied on detection of adult nematodes in the brains of WTD. The last published surveillance effort in Saskatchewan detected *P. tenuis* in the brain of one WTD harvested in the 1990s in central-east Saskatchewan, roughly 60 km west from the Manitoba border (Wasel et al., 2003). WTD populations have gradually been expanding throughout the southern boreal region over the last century due to changes in climate and land use. This has raised concerns that WTD may introduce diseases, including *P. tenuis*, to other cervid populations, such as moose and caribou, important for conservation and subsistence harvest in the boreal region of Saskatchewan (Dawe and Boutin, 2016; Barber et al., 2018). To explore this, we examined heads of harvested WTD for adult *P. tenuis* worms to determine if there have been shifts in the current western distributional limit and the degree of establishment in WTD as definitive hosts in the province. To assess the possible risk that *P. tenuis* poses to other cervid species, we also report cases of *P. tenuis* as the cause of morbidity or mortality in aberrant host species identified by the Canadian Wildlife Health Cooperative. Combining these two data sources provides stronger evidence that aberrant hosts are contracting the disease locally in Saskatchewan (vs being infected in neighbouring regions, such as Manitoba to the east, or North Dakota to the south), as moose can travel long distances and may survive at least three weeks post infection, (Lankester, 2010). We hypothesized that meningeal worm has spread further west since the last survey in the 1990s, especially at the parkland and boreal transition zones, due to northward shifts in WTD distribution and favourable climate conditions for gastropod hosts.

## 2 Methods

### 2.1 Aberrant host records

**Fig. 3:**
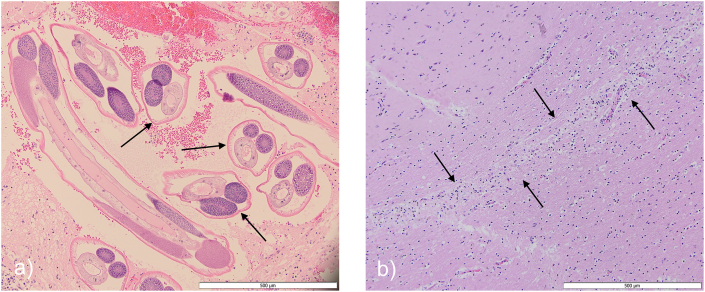
Histology from two moose infected with *Parelaphostrongylus tenuis* sampled by the Canadian Wildlife Health Cooperative between 2015 and 2024, with confirmed diagnosis based on a) the visualization of worms grossly or on histology, or suspected diagnosis based on b) characteristic histological lesions associated with nematode migration through brain tissue.

We report cases of *P. tenuis* as the cause of morbidity or mortality in aberrant host species, namely moose, mule deer, and elk found deceased or euthanized by conservation officers due to abnormal behaviour, submitted to the Western/Northern Regional Centre of the Canadian Wildlife Health Cooperative (CWHC) from 2015 to 2024. We searched the Wildlife Health Intelligence Platform (WHIP), the CWHC database, for all cervid mortalities in Saskatchewan between 2015 and 2024 where *P. tenuis* was confirmed, based on visualization of worms grossly or on histology (Fig. 3a), or suspected, based on characteristic histological lesions associated with nematode migration (Fig. 3b). Aberrant cervid hosts (moose, elk, and mule deer) were either found dead or humanely euthanized by conservation officers due to signs of illness. Individual animals underwent a standard necropsy by wildlife pathologists to determine underlying causes of disease and/or death. Brain sections were fixed in 10 % neutral buffered formalin, embedded in paraffin wax, and stained with hematoxylin/eosin prior to histological evaluation.

### 2.2 Study area and WTD sampling

In Saskatchewan, Wildlife Management Zones (WMZs) are designated for hunting and wildlife management purposes. We targeted WTD routinely harvested from WMZs within or bordering the Boreal Transition region in Saskatchewan. This ecoregion, with its upland conifer forest and abundant shrubland, provides both the parasite and the gastropod intermediate hosts with a favourable environment for transmission (Wasel et al., 2003; Vanderwaal et al., 2015). Approximately 69 % of Saskatchewan's moose population inhabits the mid-boreal upland and boreal transition ecoregions of the boreal plains ecozone (Arsenault, 2000). Furthermore, the boreal transition ecoregion borders the southern extent of the range of boreal woodland caribou, which are federally classified for conservation purposes as threatened under the Species at Risk Act (Species at Risk Act, S.C. 2002, c. 29).

We examined heads from WTD legally harvested and submitted by hunters as part of the Saskatchewan Ministry of Environment's chronic wasting disease (CWD) surveillance program. As samples were collected for purposes other than this research, University Animal Research Ethics were not required. Heads were collected in fall and early winter of 2021 (n = 51), 2022 (n = 60), 2023 (n = 100), and 2024 (n = 119) and kept frozen at −20 °C until processing the following spring. A total of 31 WMZs were sampled and the number of heads received from any one WMZ ranged from 1 to 31 over the four-year period. We excluded heads from animals that tested positive for CWD for biosafety purposes, and those from which the antlers and adjacent skull cap (generally larger males) were removed by hunters for taxidermy purposes. Sex was determined by antler presence in mature bucks. Age of WTD was estimated by visual examination using tooth eruption pattern and the degree of wear on the molar arcades (Jensen, 1996). No fawns under the age of one year were present in the dataset.

**Fig. 4:**
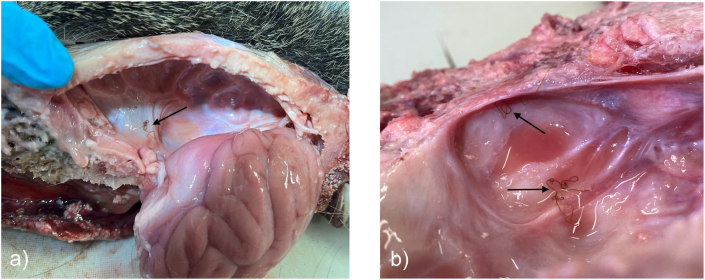
Heads from white-tailed deer (*Odocoileus virginianus*) collected for other reasons were partially skinned to expose the occipital condyles. The heads were then cut sagitally off center to preserve the venous sinuses (a). The surface of the brain as well as the cranium were examined under a light source and flushed with water to detect and recover adult worms (b).

Excess hide and tissue were removed from the neck until the occipital condyles were visible (Fig. 4a). Using a reciprocating saw, the heads were cut sagitally, from the occipital condyle to the premaxilla, off center to avoid cutting through the venous sinuses, (R. Gerhold, University of Tennessee, personal communication). Each hemisphere of the brain was gently removed, and both the surface of the brain and cranium were examined under a light source and flushed with water to remove debris and recover worms (Fig. 4b). The lateral and sagittal venous sinuses were cut open using a scalpel blade and flushed with water. Nematodes were detected by the naked eye and were compatible in size (6–9 cm long) and coloration (brown or green) with adult *P. tenuis*; subadults are rarely present in the neural parenchyma of the definitive host, as development to the adult stage occurs in the spinal cord of host (Lankester, 2001). Worms were counted as they were extracted so that segments were not double counted. The number and sex (based on uterus in females and spicules in males) of adult *P. tenuis* recovered from each WTD were recorded and worms collected in 70 % ethanol for molecular work.

### 2.3 Molecular analyses

Ethanol fixed worms were washed twice with water and DNA was extracted from the anterior half of 4 worms from 4 separate WTD for each sampling year (n = 16) using the Qiagen DNeasy Blood and Tissue extraction kit, with an initial digestion period of 1 h at 56 °C. The barcoding region of the mitochondrial Cytochrome Oxidase Subunit I (COI) was amplified in PCR using primers LCO1490 (5′ GGT CAA CAA ATC ATA AAG ATA TTG G 3′ and HCO 5′TAA ACT TCA GGG TGA CCA AAA AAT CA3’ (Folmer et al., 1994). The PCR protocol was lightly modified from Pidwerbesky et al. (2023), in which 25 μL reactions contained 10X PCR buffer, 0.75 μL of 50 mM MgCl2, 0.5 μL of each primer (10μM), 0.5 μL of 0.5 μM dNTP mix and 0.3 μL of 5 U/μL Taq DNA polymerase. The thermal profile was as follows: 94 °C for 1 min for initial denaturation, followed by 40 cycles of 94 °C for 30s, 51 °C for 40 s, and 72 °C for 1 min, with a final extension of 5 min at 72 °C.

PCR products from all amplified target regions were visualized using a 1.5 % agarose gel and PCR products that produced single bands were purified using the Qiagen QIAquick PCR purification Kit, followed by Sanger sequencing. Sequences were trimmed and aligned using CLC Main Workbench (23.0.4) and aligned along with reference sequences from GenBank and BOLD using the online version of MAFFT (Katoh et al., 2019) with default parameters. The aligned matrix was truncated in Mega7 (Kumar et al., 2016) and exported for maximum likelihood analysis in RAxML 8 (Stamatakis, 2014) using the GTRCAT approximation model. Bootstrap support (bs) was calculated using the autoMRE bootstopping function, which determines the necessary number of bootstrapping replicates needed to attain stable branch support values. We considered all bootstraps above 75 as significant. Resulting trees were visualized in Figtree 4 (Rambaut, 2018) and visually edited in Corel PaintshopPro X8.

### 2.4 Statistical analyses

A WTD was considered infected if one partial or whole adult *P. tenuis* was recovered from the surface of the brain or cranium. Subsequently, the WMZ in which it was harvested was deemed a “positive zone.” Prevalence in this study was defined as the number of WTD positive for the parasite divided by the number of samples examined x 100 %, and intensity was defined as the number of intact and partial worms detected per deer. Deer age groups were ≤1.5 years (n = 99), 2.0–3.0 years (n = 87), 3.0–4.0 years (n = 78), and ≥4.0 years (n = 66). Generalized linear models (GLMs) with binomial errors were used to analyze infection status as a function of the fixed factors age, sex, year, and WMZ. Generalized Linear Models with Poisson errors were used to analyze the intensity of infection as a function of the fixed factors age, sex, year, and WMZ. For both types of GLM, log-likelihood ratio tests were used to determine the overall statistical significance of the fixed factors. For both types of GLM, the parameter estimates were used to determine which groups were different from the reference group. Statistics were done using R (R Core Team, 2025; Posit team, 2025), and the GLMs were performed using the packages “lme4”, “car”, “MASS”, and “emmeans” (Venables and Ripley, 2002; Bates et al., 2015; Lenth, 2017; Fox and Weisberg, 2019).

## 3 Results

### 3.1 Prevalence of infection in WTD

Overall, 35 of 330 (10.6 %) WTD examined over the 4 sampling years harboured at least one *P. tenuis* adult nematode (Table 1). In total, 23 of 224 (10.3 %) male WTD and 12 of 106 (11.3 %) female WTD were positive. For the GLM with binomial errors, the ratio of the residual deviance to the residual degrees of freedom (ratio = 150.55/292 = 0.516) was 0.516 indicating that overdispersion was not a problem. Wildlife Management Zone, but not host sex (p = 0.827), year sampled (p = 0.681), or age (p = 0.549), was significantly associated with status of infection with *P. tenuis* (χ^2^ = 67.094, df = 30, p = 0.00012). Using WMZ 30 as a reference (4.2 % = 1/24), the prevalence of infection was significantly higher in WMZs 42E (66.7 % = 2/3, z = 2.181, p = 0.029), 42W (50.0 % = 1/2, z = 1.979, p = 0.0479), 50 (27.6 % = 8/29, z = 2.074, p = 0.038), 56 (50.0 % = 2/4, z = 2.096, p = 0.036), and 63 (50.0 % = 1/2, z = 2.075, p = 0.0380). WMZ 50 was the only zone positive each sampling year, with a total of eight cases over the four-year sampling period. WMZ 37 was positive for three of the four years, with a total of five cases, and WMZ 48 only became positive (5 cases) in the final sampling year.

**Table 1:** Prevalence (% positive) and intensity (number of worms per positive host) of *Parelaphostrongylus tenuis* in the heads of white-tailed deer (WTD, *Odocoileus virginianus*) submitted by hunters in Saskatchewan between 2021 and 2024.

Year	Total WTD	F:M^a^ WTD	Prevalence (%)	Number infected F WTD	Number infected M WTD	Mean (range) intensity	Total # worms	Positive Wildlife Management Zones (# +ve WTD/sample size)
2021	51	11:40	6/51 (12 %)	1	5	3.6 (1–10)	22	43 (1/4), 49 (1/3), 50 (2/14), 53 (1/4), 56 (1/2)
2022	60	22:38	4/60 (7 %)	1	3	2 (1–5)	8	37 (1/7), 50 (1/4), 59 (1/1), 63 (1/1)
2023	100	30:70	10/100 (10 %)	5	5	2.7 (1–6)	27	35 (1/5), 37 (3/11), 42E (1/1), 43 (1/4), 50 (2/5), 56 (1/1), 57 (1/1)
2024	119	43:76	15/119 (12 %)	5	10	1.4 (1–3)	21	30 (1/9), 35 (1/7), 37 (1/12), 39 (1/8), 42E (1/2), 42W (1/1), 48 (5/14), 50 (3/6), 54 (1/10)
Total	330	106:224	35/330 (10.6 %)	12	23	2.2 (1–10)	78	30(1/24), 35(2/16), 37 (5/30), 39 (1/9), 42E (2/3), 42W (1/2), 43(2/8), 48 (5/17), 49(1/5), 50 (8/29), 53 (1/29), 54 (1/25), 56 (2/4), 57 (1/1), 59 (1/1), 63 (1/2)

a F = female, M = male.

### 3.2 Intensity of infection in WTD

Overall, there was an average of 2.2 adult nematodes per WTD (range 1–10) (Table 1). For the GLM with Poisson errors, the ratio of the residual deviance to the residual degrees of freedom (ratio = 201.51/292 = 0.690) was 0.690 indicating that overdispersion was not a problem. For the intensity of infection, the GLM found significant effects of age group (χ^2^ = 8.745, df = 3, p = 0.033) and WMZ (χ^2^ = 157.769, df = 30, p < 2e-16) but not of sample year (p = 0.502) or sex (p = 0.681). Using the younger deer as the reference, the older deer in age groups 2.5 (p = 0.011), 3.5 (p = 0.021), and 4.5 years (p = 0.024) had significantly higher intensity of infection. Using WMZ 30 as a reference, the intensity of infection was significantly higher in WMZs 42E (p = 0.0002), 42W (p = 0.037), 48 (p = 0.022), 49 (p = 0.017), 50 (p = 0.001), 56 (p = 1.16e-05) and 57 (p = 0.0006). Of the 35 positive WTD, 18 had single worm infections and 17 had multiple worm infections. Of the 17 multiple worm infections, five had at least one male and female adult worms (possible breeding pair).

### 3.3 Analysis of sequenced adult worms

**Fig. 5:**
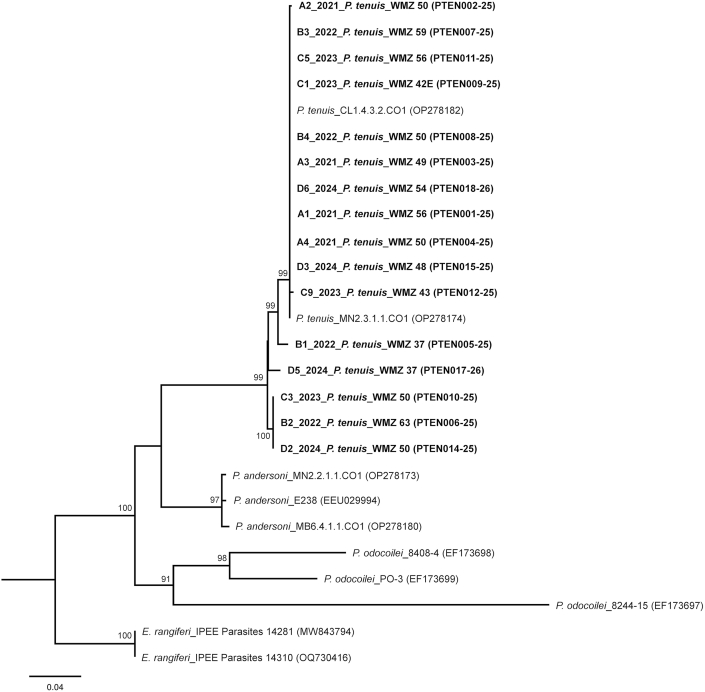
Maximum Likelihood phylogeny generated from COI sequence data. The sequences generated in this study were from adult nematodes recovered from the brains of white-tailed deer (*Odocoileus virginianus*). Sample names include year of collection, the species (*P. tenuis*), the Wildlife Management Zone (WMZ), and sequence accession numbers in BOLD. Bootstrap values > 75 are imposed on the branches as support, calculated using the autoMRE function in RAxML8.

*Parelaphostrongylus tenuis* COI sequences from Manitoba, (OP278188.1, OP278190.1, OP278193.1) were the closest match to the 16 worms sequenced, with a percent identity range of 96.6–100 %. Coupled with the phylogenetic results, our samples can confidently be identified as *P. tenuis*. The 16 sequences have been deposited in BOLD with accession numbers PTEN (001–012)-25, PTEN (014–015)-25, and PTEN (017–018)-26 (Fig. 5). Specimens fixed in 70 % lab grade ethanol have been deposited at the Royal Saskatchewan Museum in Regina, SK (Accession number 21307).

The *Parelaphostrongylus* in-group formed a monophyletic group with high support, divided into three, species-specific clades (Fig. 5). *Parelaphostrongylus tenuis* was found to be monophyletic comprising four clades, three of which have strong support. *Parelaphostrongylus andersoni* was recovered as a sister clade to *P. tenuis* with bootstrap support for the monophyly. The support for *P. tenuis* + *P. andersoni* did not reach 75 and is thus not considered significant. Finally, *P. odocoilei* was also recovered as monophyletic, sister to *P. tenuis* + *P. andersoni*.

### 3.4 Aberrant host records

**Fig. 6:**
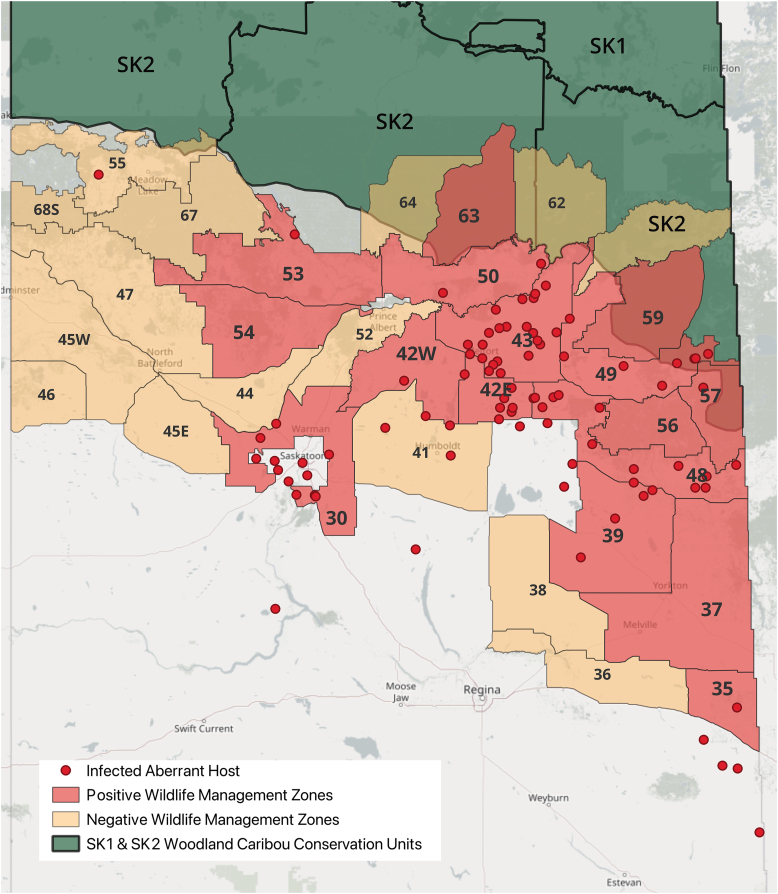
Wildlife Management Zones in Saskatchewan in which *Parelaphostrongylus tenuis* was newly detected in the heads of hunter-harvested white-tailed deer (WTD, *Odocoileus virginianus*), overlayed with dots indicating where *P. tenuis* was detected or suspected in aberrant cervid hosts between 2015 and 2024 (Canadian Wildlife Health Cooperative Western Northern), showing extensive geographic overlap. Of note, positive WTD (but not aberrant hosts) were detected in WMZs 59 and 63, and these zones overlap with woodland caribou conservation units.

Based on CWHC data, we report locations of known (47) and suspected (43) cases of *P. tenuis* detected in moose, elk and mule deer serving as aberrant hosts in Saskatchewan (Fig. 6). A significant number of WMZs had overlapping cases in aberrant hosts and in WTD (p = 0.0006), and 86 % of the 90 infected aberrant hosts came from a WMZ with at least one WTD positive for *P. tenuis*. These WMZs included WMZs 30, 35, 39, 42E, 42W, 43, 48, 49, 50, 53, and 57. Only WMZs 21, 24, 31, 32, 34, 40 and 55 were found to have aberrant host infections but not WTD infection, and only WMZs 37, 56, 59 and 63 were found to have WTD infection, but no cases in aberrant hosts (Fig. 6).

## 4 Discussion

Our results demonstrate that *P. tenuis* in white-tailed deer (WTD) has spread much further west in North America than previously described by Wasel et al. (2003) and is likely now established in northcentral Saskatchewan in both the parkland and boreal transition zones. Adult *P. tenuis* worms may live on the meninges for upwards of 4–5 years, and WTD begin to shed larvae in feces 3–4 months post infection (Duffy et al., 2002). Therefore, while the possibility that deer are contracting the parasite outside of the study area remains, five WTD in the current study were infected with both male and female nematodes and could potentially be shedding larvae of *P. tenuis* into the environment. Compared to 1990, when 1/565 infected deer was detected in one northeastern WMZ in Saskatchewan (Wasel et al., 2003), we report 36 infected deer in 15 new WMZs in the central and eastern regions of the province (Fig. 6). Our prevalence of 10.6 % is slightly higher than the 8.5 % prevalence reported in deer in North Dakota, and lower than 18.6 % reported in Manitoba in 1989–90 (Wasel et al., 2003), consistent with relatively recent range expansion into Saskatchewan from Manitoba.

Age, sex, and year were not significantly associated with infection status, as reported in other studies (Jarvinen and Hedberg, 1993; Slomke et al., 1995; Oates et al., 2000). Prevalence of infection was significantly higher (p < 0.05) in zones in the north central and eastern regions of Saskatchewan (WMZs 42E, 42W, 50, 56, and 63), consistent with more favourable moisture conditions in the parkland and boreal regions, and with range expansion from Manitoba, respectively. As sampling was limited to hunter submissions, sample distribution was highly variable, and several zones likely did not have adequate sample sizes to detect the parasite (“false negatives”). For example, *P. tenuis* was first detected in WMZ 48 in 2024, with 14 submissions; however, it is likely that *P. tenuis* had been present in WMZ 48 in previous years but was undetected due to insufficient sample numbers, as a total of three samples were submitted over the span of the first three sampling years.

While age was not a significant predictor of infection status, deer 4.5 years or older in our study had significantly higher infection intensity, likely because of greater risk of exposure over a longer time span, congruent with other studies (Thurston and Strout, 1978; Garner and Porter, 1991; Jarvinen and Hedberg, 1993; Slomke et al., 1995; Oates et al., 2000). It should be noted that in our study, fawns were not sampled. However, fawns may not be the most reliable indicator of endemic transmission since migrating worms may not reach the cranium until after their first winter (Slomke et al., 1995). No significant differences were found in infection intensity between male and female WTD, as reported in other studies (Thurston and Strout, 1978; Garner and Porter, 1991; Slomke et al., 1995).

Many environmental factors, including forest type, forest cover, soil composition and moisture, and rainfall levels are thought to impact the prevalence of infection of *P. tenuis* in cervids in North America (Wasel et al., 2003; Vanderwaal et al., 2015). The WMZs positive for *P. tenuis* in our study area were comprised primarily of boreal transition and aspen parkland ecoregions, which is favourable habitat for WTD and gastropod intermediate host populations. *Parelaphostrongylus tenuis* infection risk is positively correlated to lowland deciduous forests and upland coniferous forests found in the aspen parkland and boreal transition zones, respectively (Vanderwaal et al., 2015). Although the more arid grassland region was not examined as part of this study, it is possible that in dryer regions of province, artificial precipitation sources, such as crop irrigation, may provide enough moisture to support sufficient gastropod populations (Jacques et al., 2015).

“Microhabitat” differences, particularly forest cover, humidity, and moisture, can directly impact both gastropod population density and survival of the parasite within the environment (Wasel et al., 2003; Vanderwaal et al., 2015). As well, the definitive hosts, WTD, tend to frequent waterways, where the wetter conditions are also favourable for the gastropod intermediate hosts. Many WMZs newly positive for *P. tenuis* are intersected by, or border, large river systems in Saskatchewan, namely the South Saskatchewan, North Saskatchewan, and Assiniboine Rivers. In addition to higher densities of WTD, aberrant hosts, such as moose and mule deer, also migrate along these river systems, which may consequently increase their exposure to infection (Vanderwaal et al., 2015). The detection of *P. tenuis* in WMZs 59 and 63 is of particular importance, since these WMZs are in the southern portion of the range of woodland caribou in the province. There are no reports of infection in woodland caribou in Saskatchewan; however, there has been little sampling opportunity for this threatened species. *Parelaphostrongylus tenuis* has caused extreme population declines in caribou in eastern Canada and could also pose a significant risk to woodland caribou in Saskatchewan (Wasel et al., 2003).

The overlap between WMZs positive for *P. tenuis* through WTD sampling, and the locations of records in aberrant hosts, suggests that aberrant hosts in Saskatchewan may contract *P. tenuis* from shared environments with local deer populations. Westward and northward WTD range expansion or translocation could adversely impact populations of susceptible mule deer, moose, elk, woodland caribou, mountain caribou and wild sheep in western Canada (Jenkins et al., 2005; Leighton, 2011; Chitwood et al., 2018). While meningeal worm is often fatal in these aberrant hosts, some elk seemingly recover during experimental infection studies and go on to shed larvae (Mcintosh et al., 2007). Moose may also develop some level of immunity to the parasite; however, many infections do result in mortality, and patent infections have not been reported in moose (Lankester, 2001). Factors such as dose and acquired immunity may play a role in the severity of disease, with some hosts able to recover from low dose infections. Genetic variation within *P. tenuis* populations may also play a role in infection and host immunity; however, acquired immunity to one population of *P. tenuis* may not transfer to animals exposed to a genetically different or novel population, possibly putting translocated cervids at higher risk of infection and mortality (Chitwood et al., 2018).

While COI is demonstrated here as a reliable tool for the molecular identification of *Parelaphostrongylus* species, future studies should consider sequencing additional markers for population genetic studies that may shed light on the origin of the parasites now present in Saskatchewan.

Although some pathogens carried by WTD may pose threats to other cervid populations, WTD fulfill many important ecological roles and serve as an important prey species for larger carnivores and scavengers. Furthermore, WTD are one of the most common and widely hunted game species in North America (Edmunds et al., 2016). The rapid expansion of WTD across North America is thought to be the result of human alteration of landscapes through forestry and agriculture and the elimination or removal of natural predators. Along with natural range expansion, anthropogenic translocation of individual WTD through the cervid industry (Leighton, 2011; Dawe and Boutin, 2016) can introduce *P. tenuis* into new regions with suitable environmental conditions for its life cycle.

Our results indicate *P. tenuis* is now endemic in central Saskatchewan, including the boreal transition zone, and this may extend to near the Albertan border, based on these reports in aberrant hosts. The prevalence in Saskatchewan is lower than that reported in Manitoba and eastern Canada, consistent with a recent range expansion. Ongoing passive and active surveillance are needed to monitor trends, as *P. tenuis* occurrences in wild cervids are likely under-diagnosed. If future work demonstrates overlap in habitat usage between woodland caribou and WTD, *P. tenuis* should be considered as a potential threat to woodland caribou conservation efforts.

## 5 Conclusion

We report range expansion of the pathogenic parasite, *P. tenuis*, and its implications for the translocation of captive cervids. Our study combined active surveillance for *P. tenuis* in heads of white-tailed deer, the natural definitive hosts, with passive surveillance in aberrant cervid hosts submitted for diagnostic testing (moose, elk, and mule deer). Detection in multiple sites in central Saskatchewan supports the long-standing hypothesis that *P. tenuis* would be detected in the boreal transition zone in Saskatchewan (Bindernagel and Anderson, 1972; Wasel et al., 2003). Further efforts are needed to determine if the parasite has established further south in the prairie and shrubland ecoregions of the province. Furthermore, environmental and ecological factors driving expansion and establishment should be investigated. This work highlights the need for consideration of the potential impact that *P. tenuis* may have on cervid populations of concern for conservation and subsistence harvest in the boreal region of Canada, as well as for livestock susceptible to this pathogenic parasite.

## CRediT authorship contribution statement

**Linnea McLellan:** Writing – review & editing, Writing – original draft, Visualization, Validation, Methodology, Investigation, Formal analysis, Data curation, Conceptualization. **Trent K. Bollinger:** Writing – review & editing, Investigation, Data curation. **Gabrielle Large:** Writing – review & editing, Investigation. **Phil McLoughlin:** Writing – review & editing, Investigation, Funding acquisition. **Erin Moffatt:** Writing – review & editing, Investigation, Data curation. **Louwrens P. Snyman:** Writing – review & editing, Visualization, Formal analysis. **Iga Stasiak:** Writing – review & editing, Resources, Methodology, Investigation, Funding acquisition, Conceptualization. **Maarten Voordouw:** Writing – review & editing, Validation, Formal analysis. **Emily Jenkins:** Writing – review & editing, Writing – original draft, Supervision, Investigation, Funding acquisition, Conceptualization.

## Declaration of competing interest

The authors declare the following financial interests/personal relationships which may be considered as potential competing interests:Linnea McLellan reports financial support, equipment, drugs, or supplies, and travel were provided by Saskatchewan Ministry of Environment. Given Dr. Jenkins role on the editorial board she had no involvement in the peer review of this article and had no access to information regarding its peer review. Full responsibility for the editorial process for this article was delegated to another journal editor. If there are other authors, they declare that they have no known competing financial interests or personal relationships that could have appeared to influence the work reported in this paper.
